# Safety of a novel modular cage for transforaminal lumbar interbody fusion − clinical cohort study in 20 patients with degenerative disc disease

**DOI:** 10.1051/sicotj/2018019

**Published:** 2018-06-29

**Authors:** Mohamed Elmekaty, Emad ElMehy, Peter Försth, Anna MacDowall, Ahmed El Elemi, Mohamed Hosni, Yohan Robinson

**Affiliations:** 1 Department of Surgical Sciences, Uppsala University Hospital, Uppsala Sweden; 2 Orthopedic and Traumatology Department, Tanta University, Tanta Egypt

**Keywords:** TLIF, Cage subsidence, Large footprint, Modular cage

## Abstract

*Introduction*: Transforaminal lumbar interbody fusion (TLIF) is used to reconstruct disc height and reduce degenerative deformity in spinal fusion. Patients with osteoporosis are at high risk of TLIF cage subsidence; possibly due to the relatively small footprint compared to anterior interbody devices. Recently, modular TLIF cage with an integral rail and slot system was developed to reduce cage subsidence and allow early rehabilitation.

*Objective*: To study the safety of a modular TLIF device in patients with degenerative disc disorders (DDD) with regard to surgical complications, non-union, and subsidence.

*Methods*: Patients with DDD treated with a modular TLIF cage (Polyetheretherketone (PEEK), VTI interfuse S) were analysed retrospectively with one-year follow-up. Lumbar sagittal parameters were collected preoperatively, postoperatively and at one year follow-up. Cage subsidence, fusion rate, screw loosening and proportion of endplate coverage were assessed in computed tomography scan.

*Results*: 20 patients (age 66 ± 10 years, 65% female, BMI 28 ± 5 kg/m^2^) with a total of 37 fusion levels were included. 15 patients had degenerative spondylosis and 5 patients had degenerative scoliosis. The cages covered >60% of the vertebral body diameters. Lumbar lordosis angle and segmental disc angle increased from 45.2 ± 14.5 and 7.3 ± 3.6 to 52.7 ± 9.1 and 10.5 ± 3.5 (*p* =  0.029 and 0.0002) postoperatively for each parameter respectively without loss of correction at one year follow up. One case of deep postoperative infection occurred (5%). No cage subsidence occurred. No non-union or screw loosening occurred.

*Conclusions*: The modular TLIF cage was safe with regard to subsidence and union-rate. It restored and maintained lumbar lordosis angle, segmental disc angle and disc height, which can be attributed to the large footprint of this modular cage.

## Introduction

Degenerative disc disease (DDD) of the lumbar spine is a common cause of disability in the elderly population. Several authors found segmental instability related to DDD, thus suggesting segmental fusion for patients unresponsive to non-surgical treatment measures [1,2].

Multiple techniques have been developed to achieve segmental fusion, of which only interbody fusions maintained lumbar lordosis in a long-term follow-up [3]. All interbody fusion techniques have in common that they use the disc space for interbody fusion, but differ in approach and implant size. Anterior lumbar interbody fusion (ALIF) and oblique lumbar interbody fusion (OLIF) uses the anterior retroperitoneal approach, the extreme lateral interbody fusion (XLIF) uses the retroperitoneal transpsoaic approach, the posterior lumbar interbody fusion (PLIF) and transforaminal lumbar interbody fusion (TLIF) use posterior access to reach the interbody space [4–7].

Since it was introduced by Harms and Rolinger in 1982 transforaminal lumbar interbody fusion (TLIF) is widely utilized as an efficient procedure for achieving intervertebral body arthrodesis [8]. A TLIF cage is inserted posteriorly through a unilateral facetectomy with preservation of the contralateral facet. This reduces the iatrogenic segmental instability and minimizes the manipulation of the dura which is usually associated with (PLIF). Also, TLIF allows the reconstruction of anterior column without the complications associated with techniques using anterior or lateral approaches like accidental injury to lumbosacral plexus or intra-abdominal structures [5–8].

TLIF allows both direct and indirect decompression of affected nerve roots together with instrumentation through the same posterior approach. However, the narrow access of TLIF is insufficient for insertion of a large footprint TLIF cage to minimize cage subsidence. Therefore, loss of correction and cage subsidence is a common disturbing feature of TLIF using conventional small cages especially in osteoporotic persons [9,10].

Many biomechanical studies have reported improvement of the construct stability with expansion of the interbody cage surface area. This renders the interbody spacer based on the strong peripheral part of the vertebral endplate with subsequent reduction of cage subsidence [11–13].

Recently, an innovative modular TLIF cage has been introduced to overcome the obstacles with inserting a large TLIF cage. Its segments are small enough to be inserted through a minimally invasive access and turn into a large footprint interbody spacer after complete set-up within the disc space. Being inserted in separate modules, it minimizes bone and soft tissues destruction in addition to reducing dural manipulation which is usually associated with other conventional TLIF cages.

The aim of this study is to present the safety of a novel modular TLIF cage for patients with DDD with regard to its presumed advantages as lesser subsidence, higher union rate, and lesser surgical complications.

## Materials and methods

### Design

This is a retrospective observational cohort study on the use of a novel modular TLIF cage between 2013 and 2015 at Uppsala University Hospital (Uppsala, Sweden) with one year follow-up. This study was approved by the Uppsala regional ethical council (No. 2015-376) and reported according to CONSORT statements [14].

### Participants

Patients included in this study were operated using a novel modular TLIF cage for degenerative disc disease. Surgical intervention was considered after failure of conservative treatment for at least 6 months. Patients older than 80 years or with Body Mass Index (BMI) greater than 40, active infection or malignancy in the spinal region were excluded.

### Implants

We implanted a novel modular interbody cage (InterFuse S^™^ Intervertebral Body Fusion Device, Vertebral Technologies International, FDA 510(k) approval no. # − K093675).

**Figure 1:**
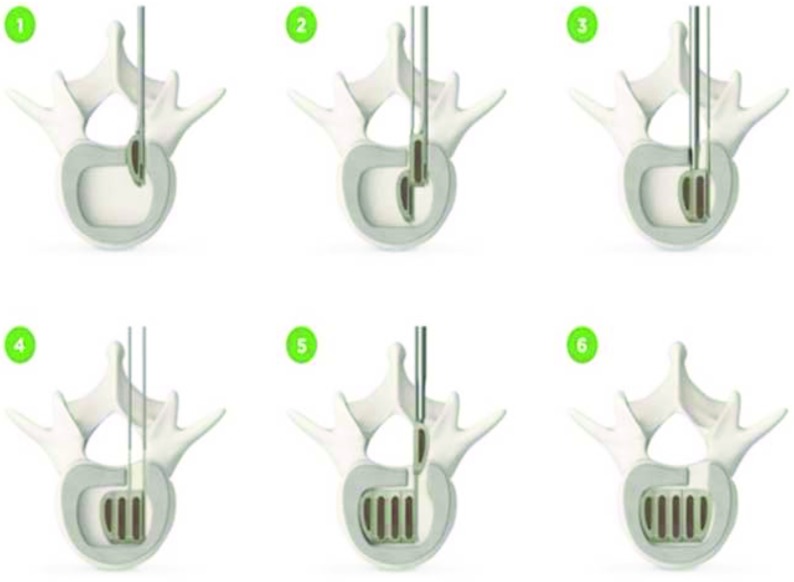
Technique of Interfuse modules assembly within disc space.

The device is made of implant grade PEEK (polyether ether ketone). It consists of an integral rail and slot multi-segmental system which is inserted through a unilateral transforaminal approach (Figure 1).

The modules are designed to lock safely to each other. It is used with autogenous bone graft and supplemental posterior spinal fixation systems. Each segment of the device has tantalum markers which allow intraoperative and postoperative assessment of cage position. We used cages with an anterior-posterior length of 20 mm, and a lordotic endplate angle of 5 degrees. The height ranges from 7 mm to 14 mm. The transverse diameter of the cage ranges from 20 mm to 38 mm according to the number of modules used per each cage (range from 3 to 6 modules).

### Cage assembly

The modular cage consists of multi segments. The A module which is the inner most module of cage is inserted firstly with its curved surface facing medially using the insertion applicator. Then, the A module is pushed medially using positioning lever and after checking the position of A module by X-ray the B module is carefully inserted by sliding the tail of A module through the distal end of the slot of the B module . Confirmation of A and B modules engagement can be checked through C-arm images. Also the tail of fully engaged A and B modules are flush with one another. The engaged A and B modules construct is pushed medially using positioning lever and the tail of a module can be removed at this stage by rotating the tail removal tool 360 degrees. Additional B modules can be inserted in the same way to maximize the foot print of cage keeping in mind leaving enough space to the outermost C module. The C module is slided along the tail of the last B module until being engaged to each other. The modules alignment could be verified by checking the marker bead location using intraoperative C-arm.

### Posterior lumbar interbody fusion using transforaminal access

To minimize inter-surgeon variability the senior author was performing or supervising all surgical interventions.

Preoperative planning was done to determine the proper cage height and number of modules to maximize the contact with the endplate and efficiently restore disc height.

The patient was placed in prone position under general hypotensive anesthesia. Through a midline subperiosteal approach Pedicular screws were inserted according to the pathology. Distraction was done at the level of TLIF. Spinal canal was entered through a limited laminectomy and medial facetectomy at the side of radiculopathy. The nerve roots are identified and retracted then complete discectomy was performed meticulously using a currete and pituitary forceps to avoid endplate breach.

The size of cage was confirmed intraoperatively by using the available trial model. The modules of the cage were filled by autograft harvested from iliac crest and were inserted sequentially and assembled within the disc space. Intraoperative fluoroscopy was used to check the position of the device. Final assembly of the screw rod system was done and the construct was compressed and tightened.

### Outcomes

We recorded adverse events and monitored them throughout the study. We considered the following as serious adverse effect:

- neurological damage related to the surgery/ implant;
- postoperative infections requiring antibiotic treatment;
- blood loss requiring transfusion;
- delayed healing;
- implant failure;
- implant malposition;
- cage subsidence.

Operation time, intraoperative bleeding amount, postoperative Serum C-reactive protein (CRP) as indicator of procedure invasiveness and hospital stay were evaluated.

**Figure 2:**
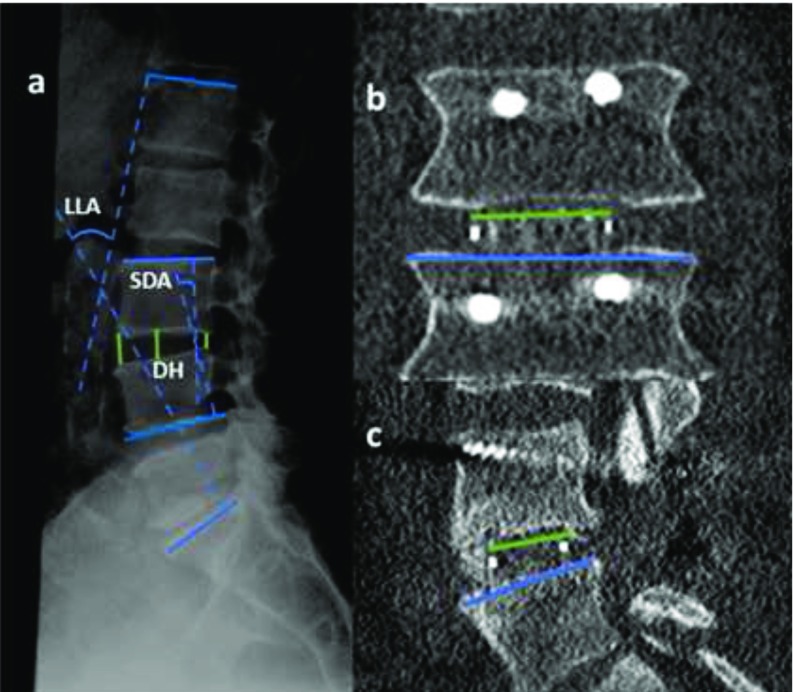
(a) Measurement of LLA, SDA and A,M,P/DH using lateral plain X-ray in a neutral position, (b,c) % CC along transverse and anteroposterior diameters.

We determined changes in lumbar lordosis angle (LLA) and segmental disc angle (SDA) using digitized radiograph analysis obtained preoperatively, postoperatively and at one year follow up. Also, changes of anterior, middle and posterior disc height (A, M, P/DH) were analyzed preoperatively, postoperatively and at one year follow- up by using lateral plain X-ray in a neutral position. Proportion of endplate coverage by the cage (%CC) was determined by measuring the dimensions of the cage guided by the tantalum beads embedded within the device in relation to the caudal endplate axis using postoperative CT scan (Figure 2).

Cage Subsidence was defined as 3 mm or more of endplate settling compared to the postoperative restoration of disc height [10]. Fusion rate, screw loosening were evaluated on follow-up investigations. Fusion was considered when there was solid consolidation within the disc space using one year follow up CT scan with absence of local mechanical instability on dynamic lateral radiograph.

All radiographical measurements were performed by the primary author using multiplanar reconstructions in Carestream PACS (Carestream Health, Stockholm, Sweden).

### Statistical analysis

All endpoints were analyzed per protocol. The Student t-test was used to analyze differences in the continuous variables. The statistical analysis was done using data analysis tool in Microsoft Excel for Mac (version 15.32, USA). A *P*-value < 0.05 was considered statistically significant.

## Results

**Figure 3:**
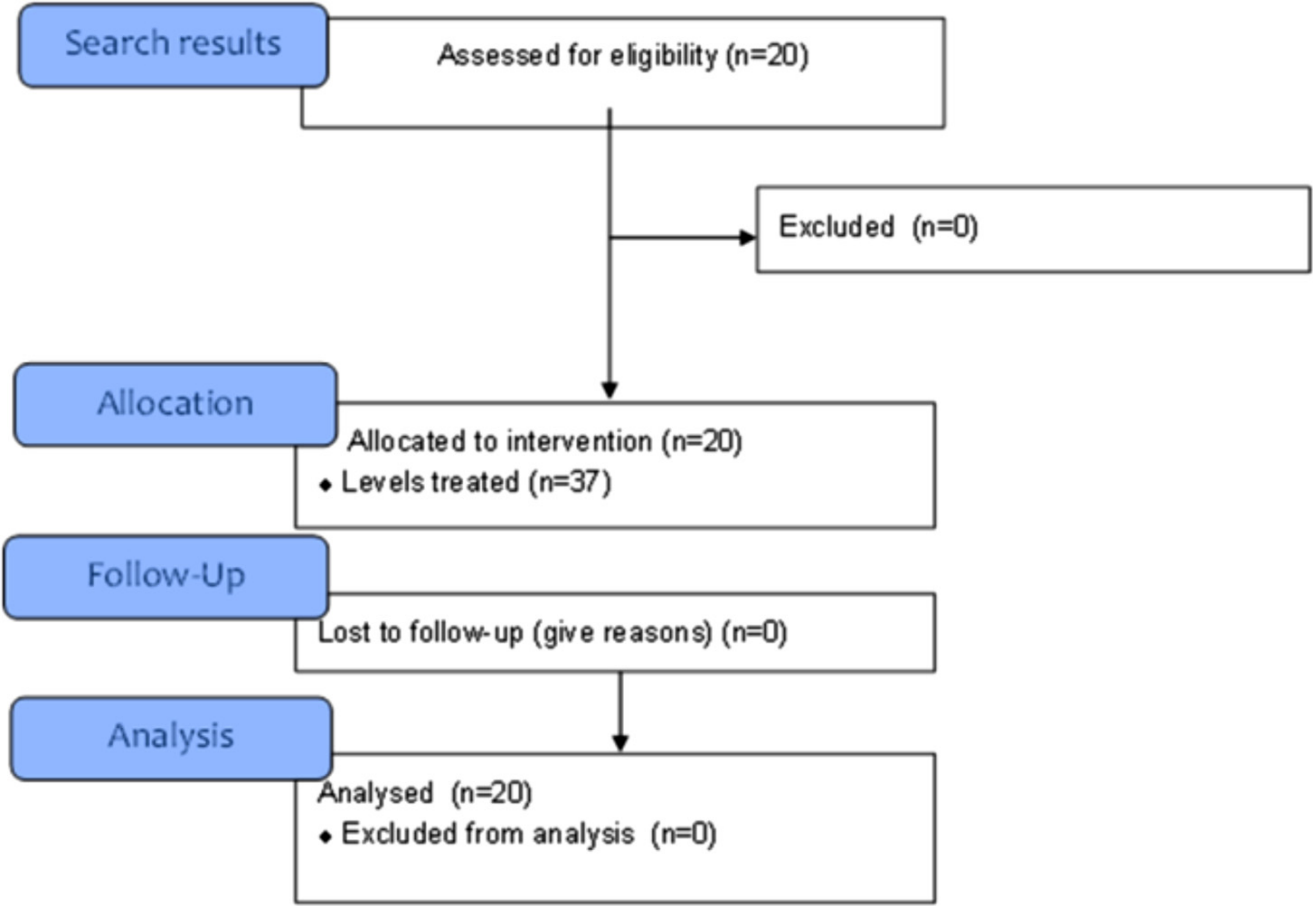
Inclusion flow diagram.

This study included 20 patients (13 female, 7 male) with average age 66.5 ± 10.2 years and average BMI 28 ± 5 kg/m^2^. The underlying pathology was degenerative spondylosis in 15 patients and degenerative scoliosis in 5 patients. All patients were followed up for a minimum of one year. The inclusion flow chart is presented in Figure 3. A total of 37 lumbar levels were fused by modular TLIF cage. The distribution of the levels of fusion was L2–L3 (6cases), L3–L4 (10 cases), L4–L5 (14 cases) and L5–S1 (7 cases).

The five multilevel deformity cases were included in the radiographical analysis, but excluded from the surgical data analysis and documented separately.

### Degenerative spondylosis surgical results

Mean operating time (166 min ± 46) while mean intraoperative bleeding amount was 1076 ± 647 mL (range: 50–2300 mL). Mean CRP at day one postoperatively was 49 ± 22 mg/L and mean hospital stay was 4.6 ± 2.1 days.

### Deformity cases surgical results

Mean operating time (358 min ± 94) while mean intraoperative bleeding amount was 368 ± 2469 ml (range:1500–7000 mL).Mean CRP at day one postoperatively was 67 ± 24.8 mg/L and mean hospital stay was 5.8 ± 2.4 days.

### Radiographical results

16.6% significant correction of LLA from 45.2 ± 14.5 degrees preoperatively to 52.7 ± 9.1 degrees postoperatively with 1.5% insignificant loss of correction at one year follow up.

4.38% significant correction of SDA from 7.3 ± 3.6 degrees preoperatively to 10.5 ± 3.5 degrees postoperatively with 6.7% insignificant loss of SDA correction at one year follow up.

Mean preoperative DH was 10.2 ± 3.4 mm, 8.8 ± 2.9 mm and 5.7 ± 1.9 mm for ADH, MDH and PDH respectively and was significantly restored postoperatively to 13.5 ± 2.8 mm, 10.8 ± 2.4 mm and 7.2 ± 1.9 mm for each parameter respectively. The mean loss of restored DH at one year follow up was 0.9 ± 0.88 mm,0.5 ± 0.9 mm and 0.6 ± 0.7 mm for each parameter respectively(*P *> 0.05). % CC endplate coverage by the TLIF cage surface was 65% and 61% along anteroposterior and transverse diameters respectively (Table 1).

**Table 1:** Radiological results.

	Preoperative	Postoperative	One year f/u	Mean restoration(%)	Mean loss of correction(%)
LLA (degrees)	45.2 ± 14.5	52.7 ± 9.1	50 ± 9.3	7.5 ± 11.5(16.6%)*	2.8 ± 2.2(1.5%)
SDA(degrees)	7.3 ± 3.6	10.5 ± 3.5	9.8 ± 3.6	3.2 ± 1.3(43.8%)**	0.7 ± 0.6(6.7%)
ADH(mm)	10.2 ± 3.4	13.5 ± 2.8	12.6 ± 2.9	3.3 ± 2.3(32,4%)**	0.9 ± 0.88(6.9%)
MDH(mm)	8.8 ± 2.9	10.8 ± 2.4	10.4 ± 2.2	2 ± 2.4(22,7%)**	0.5 ± 0.9(4.3%)
PDH (mm)	5.7 ± 1.9	7.2 ± 1.9	6.6 ± 1.6	1.5 ± 1.6(26.3%)**	0.6 ± 0.7(8.5%)

LLA - lumbar lordosis angle, SDA - segmental disc angle, ADH - anterior disc height, MDH - middle disc height, PDH - posterior disc height.

* *P* < 0.05.

** *P* < 0.01.

One rheumatoid arthritis immunosuppressed patient with degenerative scoliosis developed a postoperative deep infection. No graft's donor site related complications.

**Figure 4:**
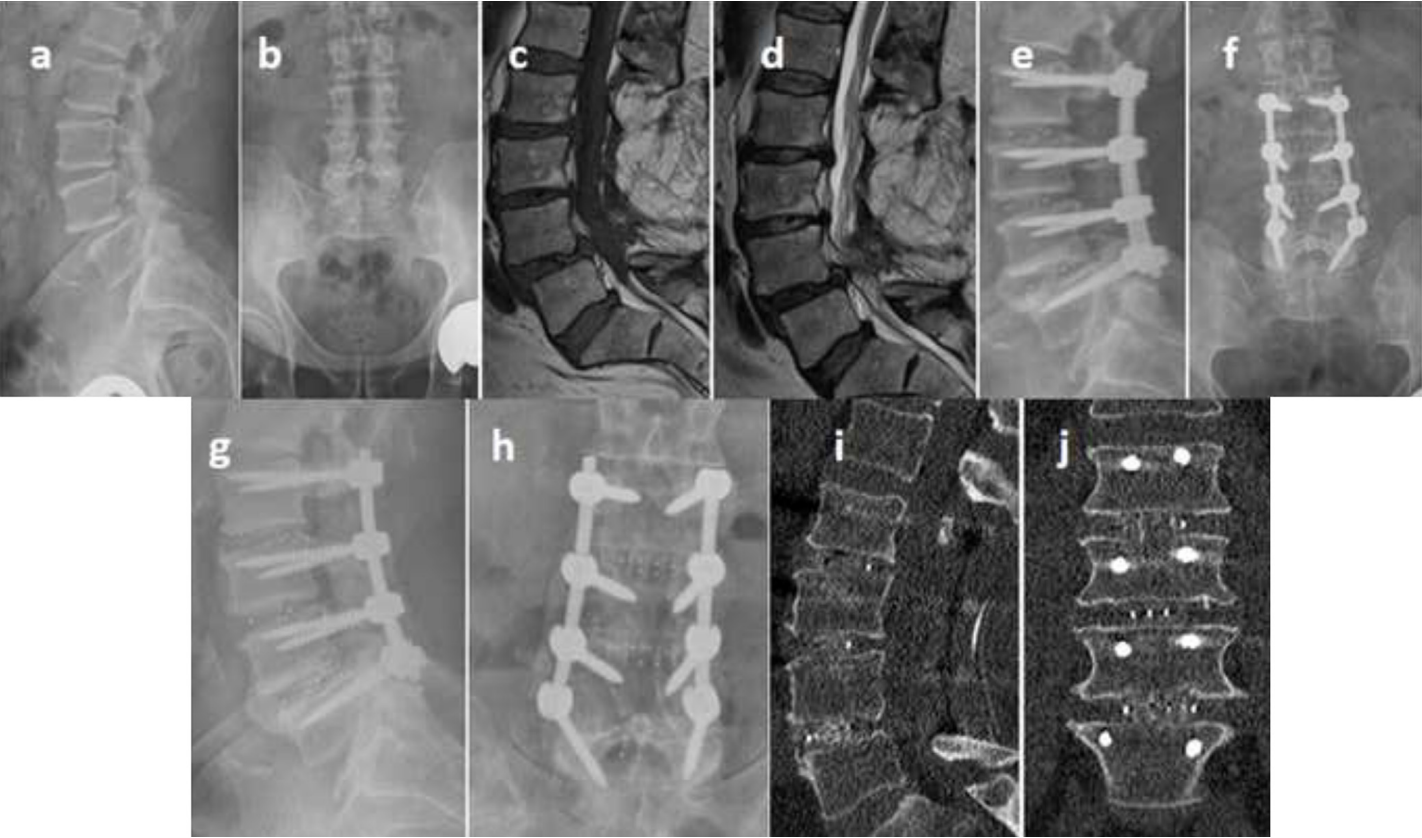
(a–d) Preoperative radiographs and MRI of 68-yrs male with postlaminectomy instability treated with TLIF L2:L5 using modular cages, (e,f) postoperative radiographs, (g–j) bony fusion was achieved at 1 year follow-up.

Radiologically 100% fusion rate at one year follow up with no loosening of screws (Figure 4).

No incidence of cage subsidence or migration. No intraoperative complications regarding cage assembly.

## Discussion

### Key findings

To the best of our knowledge, this is the first publication on the modular TLIF cage and its safety regarding serious adverse events and radiological fusion. Blood loss, infection rate, subsidence and pseudarthrosis rate were similar, or lower than reported for other TLIF implants.

### Biomechanics of TLIF with regard to subsidence

Posterior access to the interbody space allows the insertion of small footprint cages which cover the weak central part of vertebral endplate and fail to span the strong peripheral cortical ring. As the major part of applied forces transmitted through the anterior column, small cages are more susceptible to subsidence. Large footprint cages provide a stable construct which distribute these forces to wide area of end plate, with subsequent reduction of stresses applied on posterior screws [11,13,15,16].

A biomechanical analysis of a large articulating TLIF cage showed reduced incidence of its subsidence and less forces applied to posterior screws compared to a single TLIF cage model. Double TLIF model demonstrated almost similar results but it magnifies the risks during its application [17]. Also, a biomechanical study of interbody fusion graft area demonstrated significant resistance to graft subsidence when the graft is greater than 30% of the endplate surface area [18].

#### Footprint

In our study the modular TLIF cage inserted into the disc space piece by piece to form a large footprint unit to overcome the narrow access of TLIF technique. The cage covered 65% and 61% of anteroposterior and lateral diameters respectively. We reported no incidence of cage subsidence or screw loosening. Utilization of such wide based cages allowed its support by the hard peripheral ring of vertebral endplate. The supplemental posterior screws made our construct effective in correcting sagittal alignment and reestablishment of the disc height together with maintaining these changes.

Our fusion rate at one year follow up was 100%. This was due to augmenting the surface of fusion, preservation of the disc height during the healing process and converting most of applied forces by the large cage to the endplate.

#### PEEK vs. Titanium polymers

The modular cage is made of PEEK-Optima® polymer which is less stiff than titanium cages and simulates the bone young's modulus. This transmits most of the applied forces to the bone graft which promotes fusion and reduces the incidence of subsidence. Also, it facilitates the radiological assessment of fusion [19].

### Clinical importance of cage subsidence

Radiological cage subsidence is common in TLIF as its access does not allow insertion of large cages with less clinical impact as it provide both direct and indirect decompression effect of neural structures. Subsidence would be of concern for anterior and lateral access interbody fusion techniques which lack direct decompression effect. However, excessive cage subsidence compresses the neural foramen with loss of sagittal correction which could reflect on the patient overall outcome. [9,10,20]

### Invasiveness of the modular TLIF cage

Small size of the cage segments permits its insertion without the need to complete facetectomy and minimizes the invasiveness to bone and soft issue. Mean CRP values at day one postoperative in our study were less than CRP values reported by Linzer et al in their comparative study between minimally invasive posterior lumbar fusion (PLIF) 68.4 mg/L and open PLIF 72.7 mg/L [21].

The time consumed for cage assembly did not affect operation time or bleeding amount. Fritzell et al.reported in their study mean operation time 194 ± 76.8 min for instrumented interbody fusion group and mean blood loss of 1433 ± 1236 mL and 4 days mean hospital stay [22].

The safe pattern of modules assembly protects against expected complications as intraoperative and postoperative cage migration.

The patient who developed postoperative deep infection had a history of chronic rheumatoid arthritis and bronchial asthma and was under corticosteroid and previous methotrexate therapy. Debridement and vacuum assisted dressing were carried out after unresponsiveness to antibiotic treatment. Finally, revision surgery with replacement of screws and rods were done with no infection recurrence since then.

We could stratify the group according to operation time and amount of blood loss, where patients operated for degenerative scoliosis with a past history of rheumatoid disorders showed longer operation time and bleeding amount comparable to the reported outcomes of such procedures. Therefore, these patients should be adjusted in future for prospective controlled studies.

### Early reported results of modular cage

A clinical series of 104 cases managed by the modular cage reported by Butler et al. demonstrated 97% fusion rate and no cage subsidence or migration [23]. While Di Rita et al reported 68% (range 61–74%) of end plate coverage by the modular cage along the antero-posterior diameter and 55% (range 47–64%) along the transverse diameter in a 39 patients clinical study. Clinical improvement and fusion was achieved in all patients with no subsidence or migration and only one case of screw loosnening [24]. Lavelle and Tallarico used the modular cage for management of 15 patients with adult spinal deformity and excellent outcomes have been achieved in terms of 100% fusion rate, improvement of clinical outcome parameters and absence of subsidence or screw loosening [25].

### Strengths and limitations of this study

It is a retrospective analysis of a prospectively collected cohort with a small number of cases involved. Lack of control group to compare the results between various cages. Limited clinical studies reporting the outcome of the modular cage with no available biomechanical analysis of this cage. Patients included in this study had either degenerative spondylosis or degenerative scoliosis which are quite different entities. Including both in the same data is considered limitation of this study. The results of this study can be used in sample size calculations of future randomized controlled trials with subsidence as endpoint.

## Conclusion

TLIF using the modular cage demonstrated no incidence of cage subsidence or migration with high fusion rate, and no screw loosening. Also, it was effective in restoring LLA, SDA and DH and maintaining this correction. Using a large foot print cage distributed stresses to a wide area of the potent endplate periphery and provides a large surface for fusion in addition to reducing loads on posterior instrumentation. The modular TLIF-cage seems to be a safe method for interbody fusion in patients with risk of subsidence. Future studies should investigate prospectively the clinical and radiological outcome of the modular cage compared to traditional TLIF cages. Furthermore, finite element analysis of modular cage biomechanical features compared to other devices is recommended.

## Conflict of interest

ME received a travel grant by Vertebral Technologies International. EEM, PF, AMD, AEE, MH and YR had no conflict of interest.
